# Interaction Effects of Air Pollution and Climatic Factors on Circulatory and Respiratory Mortality in Xi’an, China between 2014 and 2016

**DOI:** 10.3390/ijerph17239027

**Published:** 2020-12-03

**Authors:** Kingsley Katleho Mokoena, Crystal Jane Ethan, Yan Yu, Asenso Theophilus Quachie

**Affiliations:** 1 School of Public Health, Xi’an Jiaotong University, Health Science Center, Xi’an 710061, China; crystal2016@stu.xjtu.edu.cn; 2 School of Mathematics, Northwest University, Xi’an 710127, China; theo.asenso@stumail.nwu.edu.cn

**Keywords:** interaction mechanism, meteorological condition, mortality, climatic change, distributed lag non-linear model, air pollution

## Abstract

Several studies have reported that air pollution and climatic factors are major contributors to human morbidity and mortality globally. However, the combined interactive effects of air pollution and climatic factors on human health remain largely unexplored. This study aims to investigate the interactive effects of air pollution and climatic factors on circulatory and respiratory mortality in Xi’an, China. Time-series analysis and the distributed lag non-linear model (DLNM) were employed as the study design and core statistical method. The interaction relative risk (IRR) and relative excess risk due to interaction (RERI) for temperature and Air Quality Index (AQI) interaction on circulatory mortality were 0.973(0.969, 0.977) and −0.055(−0.059, −0.048), respectively; while for relative humidity and AQI interaction, 1.098(1.011, 1.072) and 0.088(0.081, 0.107) respectively, were estimated. Additionally, the IRR and RERI for temperature and AQI interaction on respiratory mortality were 0.805(0.722, 0.896) and −0.235(−0.269, −0.163) respectively, while 1.008(0.965, 1.051) and −0.031(−0.088, 0.025) respectively were estimated for relative humidity and AQI interaction. The interaction effects of climatic factors and AQI were synergistic and antagonistic in relation to circulatory and respiratory mortality, respectively. Interaction between climatic factors and air pollution contributes significantly to circulatory and respiratory mortality.

## 1. Introduction

Ambient air pollution continues to be a dire human health threat, contributing to premature deaths in different parts of the world, more especially in the urban areas of developed and developing countries, reflecting the effects of increased urbanization, rapid industrialization, intensified use of fossil fuels, and surge in traffic volumes. Ambient air pollution comprising a complex mixture of both particles and gases remains a significant public health concern that continues to be associated with both acute and chronic health effects globally, with developing countries being the most affected [1,2]. Furthermore, exposure to ambient air pollutants has for decades been reported as a cause of both circulatory and respiratory mortality. Various studies have reviewed the literature on the epidemiological evidence of mortality (circulatory and respiratory) associated with short-term exposure to ambient air pollutants in China, where they highlighted that climatic factors played a critical role in the association between air pollution and mortality [3,4].

Climate change has the ability to influence air quality and the vice-versa, as climatic factors such as temperature, barometric pressure, wind speed, and relative humidity are known to have a large impact on air pollutants’ concentration, chemistry mixture, and how far they can be dispersed or deposited. For example, an extreme change in temperature has the ability to influence the body’s ability to regulate the internal temperature in those exposed, thereby possibly resulting in heatstroke, hypothermia, heat exhaustion, hyperthermia, frostbite, and heat cramps, all of which can result in hospitalization or even death in people with underlying illnesses such as asthma which can be triggered or worsened by poor air quality. Hence, climate change has been labeled a dire and major public health concern, with its adverse effects evidently noted in every region of the world. Subsequently, the relationship between ambient temperature and daily mortality has been extensively observed with the effect of temperature on mortality said to be influenced by various factors, including, air pollution levels, geographic location, and weather patterns, among many others [5]. Moreover, climate change (through climatic factors) and air pollution can both affect and influence the health outcomes of those exposed (directly and indirectly). Analyses conducted in Canada, Europe, China, and the United States have provided evidence of the coherence and plausibility of these associations between ambient air pollution and climate factors, suggesting a need for more studies in developing countries to ascertain the effect of ambient air pollution and climate factors on health [6,7]. However, the impact of ambient air quality and climate change on human health remains complex as there are a number of factors that come into play.

While the inter-relationship between air pollution and climate change is widely known and well documented, the recent surge in climate change has become an urgent call for concern prompting a need to understand the effect of interaction between ambient air pollution and climatic factors on health and mortality. Although both ambient air pollution and climatic factors have been closely associated with daily mortality; the effect of their interaction remains largely unknown even though first investigated in 1972 [8]. Globally, recent evidence suggests that the interaction of air pollution and climate change can significantly affect health and mortality. For instance, in Wuhan, Shanghai, and Tianjin, studies showed that extreme temperatures and relative humidity exacerbated the effect of air pollution on health [9,10,11]. Despite the growing prevalence of literature on short-term exposure to ambient air pollutants and its association with cardio-respiratory mortality in China, only megacities such as Beijing, Shanghai and Guangzhou are regularly focused on, hence, leaving a challenge in understanding the extent of the association in China [3,4]. Moreover, although there has been an increase in epidemiologic evidence of the interaction between ambient air pollution and climatic factors in recent times, studies focusing on the effect on circulatory and respiratory mortality in China (particularly in non-metropolises) remain scarce, resulting in many unanswered questions in a country with a huge burden of air pollution in the wake of climate change. With recent urbanization and mass industrialization, air pollution levels in many Chinese cities remain significantly high, exceeding the upper limits stated by both the World Health Organization guidelines and the Chinese National Ambient Air Quality Standards. Additionally, considering the acceleration of climate change and the recent exponential increase in non-communicable diseases (particularly circulatory and respiratory) in China, the interaction effect of air pollution and climate change on mortality warrants investigation.

## 2. Materials and Methods

### 2.1. Data

Located in the center of the Yellow River’s Guangzhong Plain, bordered by the Qinling Mountains to the south and Weihe River to the north, Xi’an is the most populous city in China’s northwestern province of Shaanxi, with a population of around 8.7 million residents. The city has a distinct climate pattern corresponding to the sub-humid, warm, and temperate continental monsoon climate, with four seasons annually [12]. Being the industrial epicenter of Shaanxi province, Xi’an experiences the worst air pollution in the region.

In the current study, daily mortality counts (circulatory and respiratory diseases) from the 13 districts of Xi’an between January 2014 and June 2016, were collected from the Shaanxi Center for Disease Control and Prevention (Shaanxi CDC) databases which monitors outbreaks of diseases, intending to prevent them from spreading. The mortality data-set comprised 65,535 cases reported in Xi’an during the study period and data was recorded based on the International Classification of Diseases, Tenth Revision (ICD-10), chapter IX, (Codes: I00-99 and J00-99). Daily climatological factors (temperature and relative humidity) were previously described [13]. The daily average of air quality index (AQI), and concentrations PM_2.5_, SO_2_, and O_3_, were collected throughout the study from the Xi’an Environmental Monitoring Centre. The AQI is formulated based on the level of six air pollutants, namely sulfur dioxide (SO_2_), nitrogen dioxide (NO_2_), coarse particulate matter (PM_10_), fine particulates (PM_2.5_), carbon monoxide (CO), and ozone (O_3_).

### 2.2. Statistical Analysis

#### 2.2.1. Preliminary Analysis

Descriptive statistics were carried out to give a summary of the data.

Correlation analysis: the crude correlation between ambient air pollution and climatic factors concerning both circulatory and respiratory mortality was analyzed; a scatterplot matrix was plotted to graphically reveal the correlation. Both analyses served as a preliminary analysis, to derive a well-structured statistical model.

#### 2.2.2. Distributed Lag Non-Linear Model (DLNM)

To observe the relationship between ambient air pollution and climatic factors on mortality (circulatory and respiratory), the distributed lag non-linear model (DLNM) was used Equation (1). In environmental epidemiology association studies, mortality cases on a given specific day can be affected or influenced by the delayed effects (i.e., lag effects) of environmental factors. As such, the effects of ambient air pollution and climatic factors on both circulatory and respiratory mortality were assessed using the DLNM model which accounts for these delayed effects [14] and is described below as:

(1) $$Y_{t}\sim Poisson\left(\mu_{t}\right)\;\log\left(\mu_{t}\right)=\alpha +cb\left(x_{t}\right)+ns\left(date,\;df=7\right)+\beta DOW_{t}$$

where *Y_t_* represents the daily mortality counts of circulatory and respiratory mortality confirmed on day *t*; *α* refers to the intercept; *cb*(*x_t_*) represents the cross-basis function that models the non-linear lagged effects of daily independent variables; *x_t_* represents the mean ambient temperature, and relative humidity as well as air quality index (AQI), respectively; *ns*(date, *df* = 7) represents a date variable with natural cubic spline (*ns*) to account for unmeasured time-varying confounders, long-term trends, and seasonality; 7 *df* = 7 degree of freedom; *β* represents the coefficient of corresponding terms; *DOW_t_* refers to the day of the week. The cross-basis of DLNM were used for exploring and modeling non-linear and distributed lag structure of ambient temperature, relative humidity, and air quality index effects over lag 0 to 27 [12,15]; natural cubic splines with 3 degrees of freedom were used to account for spaces of *x_t_*; spline knots were inserted at quantiles (25%, 50%, and 75%) in the log scale of *x_t_* to estimate the cumulative effects for various values of *x*.

#### 2.2.3. Poisson Regression

Prior to fitting the Poisson regression, variables (ambient temperature, relative humidity, and AQI) were dichotomized into categories based on the turning points from the DLNM model. Consequently, ambient temperature (T), relative humidity (RH) and air quality index (AQI) were transformed as *T* = 0 (if ambient temperature ≤ cut point), and *T* = 1 (if ambient temperature > cut point), *RH* = 0 and *RH* = 1, and *AQI* = 0 and *AQI* = 1. The interaction model [Equation (2)] was constructed as follows:

(2) $$Y_{t}\sim Poisson\left(\mu_{t}\right)\;\log\left(\mu_{t}\right)=\alpha +T\left(orR\right)+AQI+T\left(orR\right)\times AQI$$

Given that *AQI* is a concentration index for six criteria air pollutants, we also observed the effect of three of these air pollutants (fine particulate matter, ozone, and sulfur dioxide). These three were selected for the following reasons: PM_2.5_ is a key player in air pollution and health-related challenges, with a widespread negative impact on human health. We considered incorporating pollutants that were not highly correlated in order to derive reliable and independent estimates of the main effects of each pollutant. PM_10_ and NO_2_ were highly correlated with other pollutants and could hence lead to collinearity in the analysis. Although SO_2_ also had a high correlation with other pollutants, it was incorporated in the study in consideration of the severe haze formation experienced in the city (as SO_2_ is a major contributor to haze formation). Ozone was incorporated as the effects of the pollutant on mortality has been shown to be most prominent in the elderly, and a high percentage of the Chinese population are aged individuals [13,16].

#### 2.2.4. Interaction Analysis

To observe the effect of the interaction between ambient air pollutants and climatic factors, we stratified the effects of ambient air pollutants by temperature and relative humidity, respectively (3). To estimate interaction, the *IRR* (interaction relative risk) and *RERI* (relative excess risk due to interaction) were calculated using *RR*_11_, *RR*_01_, and *RR*_10_ estimates from the Poisson regression. In accordance with the multiplicative and additive model of interaction theory, the *IRR* and *RERI* were calculated as follows [17]:

(3) $$IRR=RR_{11}/(RR_{01}\times RR_{10})\;RERI=RR_{11}-RR_{01}-RR_{10}+1$$

Note: *RR*_01_: when *T*/*RH* = 0 and *AQI* = 1; *RR*_10_: when *T*/*RH* = 1 and *AQI* = 0; *RR*_11_: when *T*/*RH* = 1 and *AQI* = 1. No interaction was observed when *IRR* = 1 or *RERI* = 0; synergistic interaction when *IRR* > 1 or *RERI* > 0; and antagonistic interaction when *IRR* < 1 or *RERI* < 0.

All statistical analyses were carried out using *R* software (v3.1.2) (The *R* Foundation for Statistical Computing, Vienna, Austria). The Corrplot 0.84 package was used for correlation analysis and plots; while the Sandwich 2.5.1 package was used to estimate covariate matrix and confidence intervals. The glmnet 2.0-16 was used for the selection of relevant features subsequent to adding the interaction effect.

## 3. Results

### 3.1. General Status of Ambient Air Pollution, Meteorological Parameters, and Circulatory and Respiratory Mortality

A descriptive statistics summary for ambient air pollutants concentration, climatic factors, and daily circulatory and respiratory mortality counts is presented in Table 1. Based on the GB3095-2012, the annual average concentration for PM_2.5_ (66.63 μg·m^−3^) exceeded the Chinese ambient air quality standards of 35 μg·m^−3^, while O_3_ (39.62 μg·m^−3^) and SO_2_ (26.38 μg·m^−3^) concentration were below the standard of 160 μg·m^−3^ and 60 μg·m^−3^. Of the 884 days covered by the study, daily PM_2.5_ concentrations exceeded the daily limit of 75 μg·m^−3^ for 232 days; O_3_ and SO_2_, however, did not exceed the daily limits of 160 μg·m^−3^ and 150 μg·m^−3^, respectively. For the meteorological parameters, the daily averages for temperature and relative humidity indicates that the study period had a relatively cool and moist atmosphere. The average AQI suggests Xi’an city was lightly polluted (light pollution AQI ranges between 101–150) during the study period. However, only 208 days observed the exact light pollution AQI range; while 136 days observed concentrations above this range and 540 days noted concentration levels below this range.

### 3.2. Correlation between Air Pollutants and Climatic Factors

**Figure 1:**
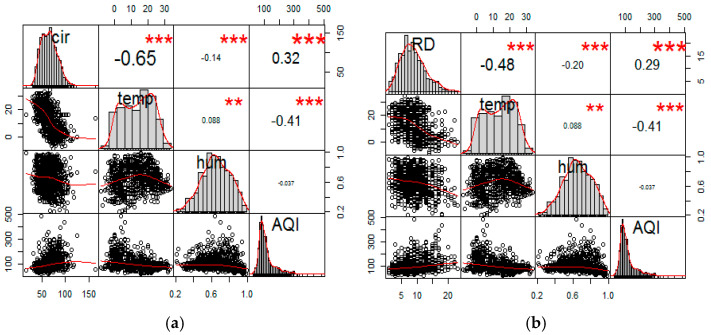
Scatterplot matrices of correlation between AQI, temperature, relative humidity, and both mortality (circulatory and respiratory) in Xi’an (2014–2016). (**a**) Circulatory mortality. (**b**) Respiratory mortality. Cir: Circulatory mortality; RD: Respiratory mortality; AQI: Air quality index; Temp: Temperature; Hum: Relative humidity; ** *p* < 0.01; *** *p* < 0.001.

The correlation coefficients between all variables are presented in Table 2 and Figure 1. Spearman’s correlation was used to demonstrate the correlation between the variables of interest. Of the three air pollutants analyzed, PM_2.5_ and SO_2_ had positive correlations with each other; however, a negative correlation was observed between O_3_ and other ambient air pollutants. Temperature showed negative correlations with other ambient air pollutants, except for O_3_ which showed a high positive correlation. Relative humidity was negatively correlated with all other pollutants except PM_2.5_.

**Figure 2:**
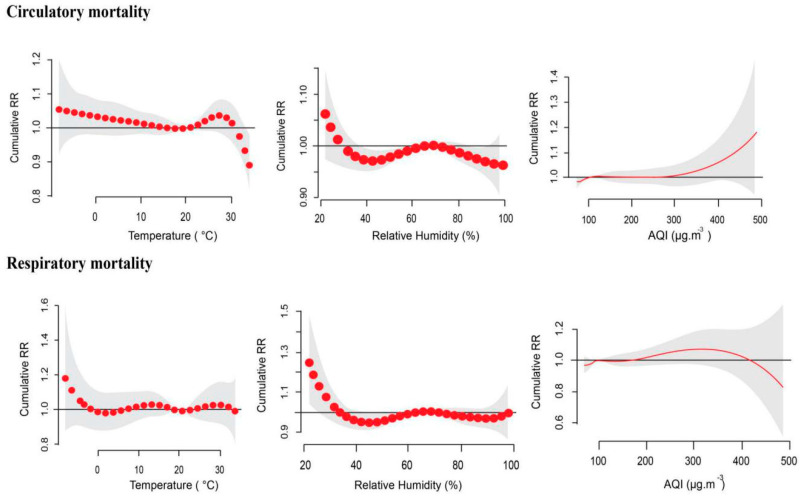
The cumulative effect of climatic parameters (temperature (°C), relative humidity (%), and AQI (µg·m^−3^) in relation to mortality (circulatory and respiratory).

The results below (Figure 2) were generated to observe the correlation between air quality index and climatic factors (temperature and relative humidity) and mortality (circulatory and respiratory). The correlation coefficients and scatterplots matrix shows that temperature (*r* = −0.65; *r* = −0.48) and relative humidity (*r* = −0.14; *r* = −0.20) were negatively correlated with both circulatory and respiratory mortality, respectively (*p* < 0.001), while AQI was positively correlated with circulatory (*r* = 0.32, *p* < 0.001) and respiratory mortality (*r* = 0.29, *p* < 0.001), respectively. However, AQI was negatively correlated with temperature (*r* = −0.404) and relative humidity (*r* = −0.037), while temperature and relative humidity had a positively significant correlation (*r* = 0.088, *p* < 0.001).

### 3.3. Correlation between Air Quality Index and Climatic Factors on Mortality

The scatter plots and matrices of correlation coefficients between AQI, temperature, relative humidity, and mortality (circulatory and respiratory) are presented in Figure 1.

### 3.4. Interaction Effects of Air Pollution and Climatic Factors on Mortality

Figure 2 displays the cumulative exposure–response curves of climatic parameters (temperature and relative humidity) and AQI in relation to circulatory and respiratory mortality in Xi’an over 30 days lag. For climatic parameters, the figure displays the cumulative relative risk that is associated with an increase in air pollution concentration (AQI), temperature, and relative humidity over a period of time. The bold red line indicates the precise relative risk estimate for exposure to predictor variables, while the gray shaded area highlights a range within which increased risk is still associated with exposure to the predictor variable. In other words, the gray area represents the lower and upper bound of the relative risk interval. The exposure–response relationship of relative humidity and mortality followed a similar trend (i.e., high relative risk for relative humidity between 20–35%, followed by reduced risk from ~36–60% RH, a slight increase in risk from ~61–75% relative humidity and a decrease from 765–100% relative humidity; in the case of respiratory mortality, a slight increase was observed from 95% relative humidity). AQI, however, displayed different trends for its cumulative exposure–response relationship with circulatory and respiratory mortality, respectively. Nonetheless, a narrow relative risk interval was noted for both types of mortality in relation to exposure of air pollution concentration between <100–250 µg·m^−3^; while a very broad relative risk interval was observed for exposure to concentration >400 µg·m^−3^. The cumulative relative risk associated with temperature exposure showed a slightly different response trend for each mortality, although a distinct similarity (no association at 20 °C, RR = 1) was observed for both types of mortality.

Table 3 and Table 4 show the mortality risk that is associated with the interaction between air pollution and climatic factor at the set cut-off point. Additionally, the interaction relative risk (IRR) and relative excess risk due to interaction (RERI) for both types of mortality are also presented. The interaction between temperature and RH and AQI in relation to circulatory mortality were all statistically significant (*p* < 0.05). However, the resultant interactive relative risk (IRR) estimate indicated that temperature and AQI interactions were antagonistic (IRR < 1, RERI < 0), while RH and AQI interactions were synergistic (IRR > 1, RERI > 0). For respiratory mortality, the interaction T = 1, AQI = 1 and H = 1, AQI = 1 regressors were not statistically significant (*p* < 0.05). Similar to circulatory mortality, the IRR and RERI suggested that temperature and AQI interactions were antagonistic while RH and AQI interactions were synergistic.

To further understand the synergistic and antagonistic relationship between the predictor variables, the Glmnet analysis results are presented in Table 5. The results indicate the actual contribution or fit of each predictor variable to the model estimating circulatory and respiratory mortality risk.

Table 5 shows the interaction coefficient between climatic factors (temperature and relative humidity) and air pollution (AQI, PM_2.5_, O_3_, and SO_2_). The main or individual effects, as well as the combined or interactive effects, are reported to distinguish which parameters are synergistic and which are antagonistic.

Since the temperature was observed to modify the contributions of all air pollutants on circulatory and respiratory mortality, the risk associated with the temperature at its minimum and maximum, in addition to the air pollutants, were analyzed in order to establish the cold (−8 °C) and hot (34 °C) effect on both mortality categories (Table 6).

## 4. Discussion

Climate factors such as temperature and relative humidity play a pivotal role in determining the distribution, patterns, and concentrations of air pollution in our environment at any given time. Thus, climate factors and air pollution are closely associated with human health. However, these parameters are influenced by environmental and geographic factors; as such, their actions vary to a certain degree, thereby creating a need to fully understand their associations, interactions, and how they are likely to contribute to health and mortality in different regions. This study explored the basic and complex relationship between meteorological parameters and air pollution, in a bid to understand their effect on circulatory and respiratory mortality. The preliminary analysis (descriptive statistics and correlation) revealed basic but vital information about each variable. The descriptive statistics highlighted PM_2.5_ as the only air pollutant that exceeded both its daily and yearly limits, hence revealing the severity of PM_2.5_ emission in the city; a finding that has also been established by other ecological studies [18,19]. For the meteorological parameters, the averages suggest that the study period was relatively cool and moist; nonetheless, this does not imply mortality (circulatory or respiratory) was associated with cold temperatures or moist atmospheric condition only. It is important to note that both cold and hot temperatures have differing effects on circulatory and respiratory mortality. As seen in Figure 2, increased relative risk was observed at both high and low temperatures for each mortality, while at low and average percentages of relative humidity, increased relative risk was observed for both types of mortality, although the risk associated with low temperature and relative humidity surpasses the risk associated with higher temperature and relative humidity. The effect of cold temperature has been known to increase cardiovascular strain in healthy individuals via physiological reactions targeted to maintain heat balance. These reactions may be exacerbated in individuals with cardiovascular conditions involving altered nervous system, cardiac and circulatory function [20,21]. Low relative humidity, on the other hand, has been associated with increased loss of water from the body, through the skin as well as the mucus membrane (in an attempt to moisten air flowing to the lungs). The less humid the air, the more moisture the body attempts to put into the air flowing to the lungs, subsequently resulting in a strain on the respiratory tract and system [22]. The average AQI concentration in Xi’an was 105 µg·m^−3^, which is within the slightly polluted index range. This pollution level has been considered a health threat for immuno-compromised people, suggesting people with underlying circulatory and respiratory diseases were at an increased risk of mortality when exposed to such air pollution concentrations. The positive correlations observed indicate that increment per unit of one variable was directly proportional to increment of the other variable being correlated (revealing if collinearity exists between said variables). For instance, the significant positive correlation observed between O_3_ and temperature suggests that, as temperature increases, O_3_ concentration levels also increase. This was shown to be accurate as ozone levels were noted to be high during the hot seasons (graphically displayed in our previous study) [13,16]. On the other hand, a negative correlation indicates two variables are inversely proportional; therefore, the negative relationship between circulatory mortality and temperature denotes that, as temperature increases, circulatory mortality decreases. However, this will only be completely correct if the relationship is a perfectly linear one; however, all bivariate relationships are not linear. Hence, the correlation analysis was used to reveal relationships (not associations) between variables, which was advantageous in structuring the model of the study.

Evidence suggests that interaction between air pollution and climatic factors exist and may have an effect on mortality, a revelation which is a major concern considering accelerated global climate change, a recent sharp increase in non-communicable diseases, and exponentially increased levels of air pollution, particularly in China. Patterns of temperature and mortality have been presented by various studies with U, V, and J curves [12,23,24]. The cumulative effects of temperature in Xi’an presented a typical U-shaped exposure-response curve in relation to circulatory mortality, whereas respiratory mortality was a reversed J-shape. This suggested that circulatory mortality was mostly higher during extreme cold and warm days, an observation that has been previously reported by Tian et al. in their Beijing study, where they noted that cold effects can last for weeks in comparison to hot effects which only last for a few days [25]. With regards to respiratory mortality, the reversed J-shaped exposure-response association with temperature suggest that colder periods were responsible for a substantial fraction of respiratory mortality [26]. This outcome was consistent with Gasparrini et al., whose findings stated that cold temperatures were responsible for a substantial percentage of deaths compared to hot days [27]. On the contrary, the outcome was the opposite of that of Pinheiro et al. who reported elevated mortality at high temperatures (i.e., J-shaped association curve) [28]. Various studies have noted cold and warm temperatures as having adverse health effects that could potentially lead to fatality in people with different diseases (including circulatory and respiratory diseases) [25,29]. Generally, relative humidity at high levels is known to diminish the body’s ability and effectiveness in transporting metabolic heat; while at low levels, relative humidity leads to dehydration, all of which could be fatal [30]. Notwithstanding this physiological and biological importance, relative humidity remains rarely examined independently in health and environmental factors association studies, except as a confounding variable. Consequently, the lack of studies investigating the role of relative humidity on health can potentially underestimate its effect, particularly with the continued rise of climate change. In the current study, relative humidity was closely associated with both circulatory and respiratory mortality at lower ranges, suggesting that mortality was high during less humid days. The findings were in agreement with Barreca, who reported that the effects of relative humidity on mortality were more common in cold counties in the United States than in hot counties [31]. With the respiratory system easily exposed to the outside environment, in Taiwan relative humidity at lower levels was closely associated with chronic obstructive pulmonary disease exacerbation during cold winter times, a potentially fatal scenario [32]. With regards to AQI, a J-shaped association was noted between AQI and circulatory mortality signifying that higher AQI was directly proportional to mortality [33]. Similarly, an increase in AQI was associated with an increase in respiratory mortality; however, an increase in AQI beyond 350 was shown to be associated with a lesser risk of respiratory mortality. The exact reason for this was not established. However, the fact that ecological-health-related relationships do not always follow a linear trend explains a great deal. Most such relationships are non-linear; hence they often show a varying pattern of exposure–response rather than a straight line [34].

Generally, the effects of interactions between AQI and climatic factors on circulatory mortality have a relationship. In relation to circulatory mortality, the RR (95% CI) for the interaction terms for temperature and humidity (T = 1, AQI = 1 and RH = 1, AQI = 1) were observed at 0.843(0.782, 0.909) and 1.126(1.037, 1.223), respectively. Additionally, for temperature, the IRR and RERI for the interaction in relation to circulatory mortality were recorded as 0.973(0.969, 0.977) and −0.055(−0.059, −0.048), respectively; while the IRR and RERI for relative humidity were 1.098(1.011, 1.072) and 0.088(0.081, 0.107), respectively. With regards to respiratory mortality, the RR (95% CI) for interaction terms for both for temperature and humidity (T = 1, AQI = 1 and RH = 1, AQI = 1) were 0.784(0.613, 1.003) and 1.069(0.913, 1.253), respectively. Furthermore, the IRR and RERI for the interaction in relation for respiratory mortality were 0.805(0.722, 0.896) and −0.235(−0.269, −0.163) for temperature, and 1.008(0.965, 1.051) and −0.031(−0.088, 0.025) for relative humidity. These results suggest that temperature did not interact with AQI to improve or influence the individual effect of AQI (i.e., an antagonistic relationship). However, taking into consideration that the air quality index represents the concentration level of six criteria air pollutants (some of which do not typically correlate positively with temperature), the observation of temperature’s interaction with individual air pollutants was paramount. A number of studies have highlighted the outcome of PM_2.5_ and temperature’s interaction on human health; Kioumourtzoglou et al. observed a higher association between long-term PM_2.5_ exposure and mortality in warmer cities; Imaizumi et al. noted a coexistence of low temperature and high PM_2.5_ was associated with a 2.3-fold increased likelihood of morning hypertension. [35,36]. Additionally, analyzing the main effects and interaction effects of individual air pollutants gave a holistic view of air pollution’s effect on respiratory and circulatory mortality. Based on the findings of this study, the interactions between climatic factors and air pollutants are proven to contribute relevantly to changes in both circulatory and respiratory mortality. However, from the two climatic parameters observed, temperature interactions with individual air pollutants were more positive in leading to an increase in mortality, as compared to relative humidity. Therefore, the risk associated with the temperature at its minimum (−8 °C) and maximum (34 °C), were analyzed in order to establish the cold and hot effect on both mortality categories. The results revealed that both cold and hot temperatures were associated with an increased risk of circulatory and respiratory mortality [37,38,39]. At its minimum, the effect of temperature was statistically significant for both circulatory and respiratory mortality with estimates 1.745(0.547, 5.567) and 10.652(0.987, 11.501) respectively, while at its maximum the effect of temperature was statistically significant for circulatory mortality only.

In comparing the risk associated with cold effects on both types of mortality, the estimates clearly show there is a higher risk of respiratory mortality due to very low temperature than circulatory mortality [40]. While hot and cold effects are evidently associated with circulatory mortality, the risk associated with extreme cold is slightly higher. These findings are supported by the fact that the respiratory organs are usually burdened by illnesses during cold seasons and the circulatory organs are mostly stressed and overworked during the hot seasons [41,42,43]. Given that these temperatures do not last for only a single day but might stretch out over several days and their effects might not be observed on the initial day of exposure, their delayed effects were analyzed over 30 lag days [44]. The results of the current study show that both cold and hot effects on circulatory and respiratory mortality were harvested at different lags [44,45]. The cold effect of temperature was significantly associated with the risk of circulatory and respiratory mortality at lag 7 [40], while the hot effect was significantly associated with risk of circulatory mortality at lag 0 and 21 and with respiratory mortality at lag 7 [46]. Studies in and out of China have related cold and hot effects of temperature to circulatory and respiratory mortality, with most revealing somewhat similar findings to the ones presented herein [47,48,49,50,51]. A study in Serbia reported heat-related cardio-respiratory mortality was maximum on the same day of exposure or lags 1–3, while cold spells were distributed over lag 3–6 [40]. Similar to our study, hot effects were observed on the current day of exposure as well as at lag 7 for circulatory mortality, while the cold effect was observed at lag 7 for respiratory mortality. They also reported the highest relative risk for mortality noted in their study was attributed to the cold days, which is also in line with the relative risk associated with respiratory mortality in this study [40]. Gasparrini et al. performed a multi-city study in several Asian and western countries and showed the effect of cold and hot temperatures on mortality, with cold temperature having the most attributed temperature-related deaths, which corresponds to the findings of our study [28,52,53].

The regression analysis carried out generated results revealing the main effects and interaction effect of climatic factors and AQI. The results from the analysis prove that climatic factors and AQI are relevant variables in predicting expected changes in both circulatory and respiratory mortality. The regression coefficients showed each variable and the interaction term contributed to changes in the outcome of interest (circulatory and respiratory mortality). They also indicate the direction of the contribution, that is positive or negative. For both mortality categories, AQI had a positive coefficient, while the climatic factors had negative coefficients. The interaction between climatic factors and AQI had a positive coefficient for circulatory mortality only. It is important to note that a negative coefficient of a variable does not equate to the non-relevance of the variable. It only reveals the less contributory factor to the outcome. Looking at the main effects of each variable, it is safe to say that a unit increase of temperature (°C) and relative humidity (%) would increase circulatory and respiratory mortality by −0.216% and −0.164%, respectively. In other words, per unit increase of this climatic factor both mortalities decrease by the percentage given above. This finding corresponds accordingly with the correlation analysis, which shows both climatic factors were not directly proportional with both mortalities. The regression coefficient and correlation also suggest that, as the climatic factor decreases, mortality increases. The positive coefficients of AQI indicate that the variable contributes 0.005% and 0.062% to circulatory and respiratory mortality per unit (µg·m^−3^) increase. The interaction term which had a positive coefficient for circulatory mortality only indicates that the interaction between climatic factor and AQI was synergistic, given that the interaction effect of the variables on circulatory mortality was greater than the main effects of each variable on circulatory mortality. In regard to respiratory mortality, the interaction effect was antagonistic especially in consideration of AQI’s main effect. This is so since the main effect of AQI was positive, contributing 0.062% to respiratory mortality; however, interaction with climatic factors changed the main effect of AQI, resulting in a negative contribution of −0.019%. All variables and interactions were shown to have contributed to circulatory and respiratory mortality with the exception of (Humidity × Ozone). The interaction term—(Humidity × Ozone) indicates that no contribution (negative or positive) was noted from the pair. However, it is important to note that the main (sole) effect of O_3_ was observed to contribute 0.0005% to respiratory mortality for every 1 µg.m^−3^ increase, suggesting that humidity interacted antagonistically with O_3_ in relation to respiratory mortality. On the other hand, effect of O_3_ interaction with temperature on both types of mortality was synergistic, contributing 0.070% and 0.018% change in circulatory and respiratory mortality, respectively, per unit increase. PM_2.5_ main effect and interaction effect with temperature contributed positively to changes in both types of mortality, while its interaction with humidity contributed positively to changes only in respiratory mortality. The main effect of SO_2_ was positive for respiratory mortality only; its interaction with temperature and humidity was however positive for circulatory mortality. This reveals that the interaction of climatic factors and SO_2_ on circulatory mortality leads to increased changes in mortality, rather than the sole effect of SO_2_. In all the air pollutants observed, SO_2_ contributed the highest to respiratory mortality with 0.125% in terms of the main effect, while PM_2.5_ contributed the highest to circulatory mortality with 0.036%. With regards to interaction effect, temperature interaction with O_3_ contributed the most to the increase in circulatory mortality, while humidity interaction with PM_2.5_ contributed the most to respiratory mortality.

### Strengths and Limitations

To the best of our knowledge, this study is the first of its kind in China to explore the interaction of air pollution and climatic factors on cardiovascular and respiratory mortality. It highlights major contributors in the relationship between air pollution and cardiovascular and respiratory mortality in Xi’an. Results for both main and combined effects strengthen the awareness that climatic conditions play a vital role in exacerbating or limiting the effect of air pollution on human health. However, the study was limited in one aspect; it lacked the stratification of mortality data into common subgroups.

## 5. Conclusions

In summary, the scenario of the interaction between air pollution and climatic factors as well as its effect on circulatory and respiratory mortality is complex, with a variety of dynamics to be considered. The current study deduced the following; air pollution and climatic factors had both individual and interactive effects on circulatory and respiratory mortality in Xi’an. Low relative humidity and temperature were associated with a slightly higher risk of circulatory and respiratory mortality than their high counterparts. The effects of interaction differ based on the health outcome; as observed, temperature noted a synergistic relationship with individual air pollutants, rather than AQI, with regards to both types of mortality, while relative humidity noted a synergistic relationship with AQI (for both mortality), PM_2.5_ (for respiratory mortality), and SO_2_ (for circulatory mortality). These findings suggest that the observation of air pollution should often consider index concentration as well as individual pollutant concentration. Finally, understanding the interaction effects of air pollution and climatic factors on mortality is essential in establishing effective mitigation and adaptation strategies to reduce circulatory and respiratory mortality in China; hence more studies should investigate these relationships in different geographical locations.

## Figures and Tables

**Table 1:** Descriptive statistics of ambient air pollutant concentrations, climatological factors, and mortality (cardiovascular and respiratory) in Xi’an from 2014–2016.

Variable	Mean ± SD	Min	P25	P50	P75	Max
Air pollutant						
PM_2.5_ (µg·m^−3^)	66.63 ± 50.13	11	36	53	77.25	527
SO_2_ (µg·m^−3^)	26.38 ± 21.55	2	12	19	35	145
O_3_ (µg·m^−3^)	39.62 ± 24.45	5	20	33	56	117
AQI (µg·m^−3^)	105 ± 55.77	19	71	89	119	486
Meteorological parameters						
Temperature (°C)	13.48 ± 9.60	−8	5	14	22	34
Humidity (%)	65.43 ± 15.8	21	55	65	78	99
Mortality						
Circulatory (*n =* 57,570)	65.09 ± 17.01	16	52	64	76	164
Respiratory (*n =* 7965)	9 ± 3.95	1	6	9	11	23

^a^ 24-h averages for fine particulates (PM_2.5_,) and sulfur dioxide (SO_2_); the maximal 8-h average for O_3_; AQI: Air quality index; SD: Standard deviation; Px:xth percentiles; Min:Minimum; Max:Maximum.

**Table 2:** Spearman’s correlation coefficients between daily ambient air pollutants and climatological factors in Xi’an from 2014–2016.

Variables	SO_2_	O_3_	Temperature	Humidity
PM_2.5_	0.670 **	−0.441 **	−0.431 **	0.041
SO_2_		−0.666 **	−0.786 **	−0.248 **
O_3_			0.778 **	−0.240 **
Temperature				0.058

** *p* < 0.01.

**Table 3:** Interactive analysis between AQI and climatic factors on circulatory mortality.

Variables	Regressor	Estimates	*p-*Value
Temperature (°C)	T = 1, AQI = 0	0.764(0.739, 0.789)	<0.001
	T = 0, AQI = 1	1.134(1.091, 1.179)	<0.001
	T = 1, AQI = 1	0.843(0.782, 0.909)	<0.05
IRR		0.973(0.969, 0.977)	
RERI		−0.055(−0.059, −0.048)	
Relative humidity (%)	RH = 1, AQI = 0	0.909(0.875, 0.943)	<0.001
	RH = 0, AQI = 1	1.129(1.055, 1.209)	<0.001
	RH = 1, AQI = 1	1.126(1.037, 1.223)	<0.001
IRR		1.098(1.011, 1.072)	
RERI		0.088(0.081, 0.107)	

AQI: Air quality index; IRR: interaction relative risk; RERI: relative excess risk due to interaction; T: temperature; RH: relative humidity.

**Table 4:** Interactive analysis between AQI and climatic factors on respiratory mortality.

Variables	Regressor	Estimates	*p-*Value
Temperature (°C)	T = 1, AQI = 0	0.799(0.749, 0.852)	<0.001
	T = 0, AQI = 1	1.220(1.133, 1.314)	<0.001
	T = 1, AQI = 1	0.784(0.613, 1.003)	0.273
IRR		0.805(0.722, 0.896)	
RERI		−0.235(−0.269, −0.163)	
Relative Humidity (%)	RH = 1, AQI = 0	0.845(0.792, 0.902)	<0.001
	RH = 0, AQI = 1	1.256(1.096, 1.439)	<0.001
	RH = 1, AQI = 1	1.069(0.913, 1.253)	0.266
IRR		1.008(0.965, 1.051)	
RERI		−0.031(−0.088, 0.025)	

AQI: Air quality index; IRR: interaction relative risk; RERI: relative excess risk due to interaction; T: temperature; RH: humidity.

**Table 5:** Coefficients of interaction between climatic factors and air pollution in relation to circulatory and respiratory mortality.

Variables	Circulatory Mortality	Respiratory Mortality
Temperature	−0.222	−0.016
Relative humidity (RH)	−0.25	−0.058
AQI	0.005	0.062
PM_2.5_	0.036	0.027
O_3_	−0.023	0.0005
SO_2_	−0.082	0.125
Temp × PM_2.5_	0.014	0.020
Temp × O_3_	0.070	0.018
Temp × SO_2_	0.020	−0.026
RH × PM_2.5_	−0.031	0.060
RH × O_3_	−0.030	0.000
RH × SO_2_	0.059	−0.072
Temp + RH × AQI	0.0524	−0.019

**Table 6:** Temperature effects at the minimum (−8 °C) and maximum (34 °C) average temperature.

Temperature (°C)	Circulatory Mortality	Respiratory Mortality
**−8 °C**	1.745(0.547, 5.567)	10.652(0.987, 11.501)
−8 °C Lag 0	0.967(0.824, 1.136)	0.921(0.672, 1.263)
−8 °C Lag 7	1.011(0.955, 1.071)	1.056(0.942, 1.185)
−8 °C Lag 14	1.024(0.976, 1.074)	1.098(0.996, 1.210)
−8 °C Lag 21	1.038(0.990, 1.088)	1.137(1.032, 1.252) *
−8 °C Lag 28	1.004(0.944, 1.068)	1.067(0.937, 1.215)
**34 °C**	2.471(0.916, 6.662)	0.887(0.117, 6.689)
34 °C Lag 0	1.275(1.116, 1.458) *	1.177(0.888, 1.556)
34 °C Lag 7	0.958(0.906, 1.013)	0.951(0.848, 1.067)
34 °C Lag 14	1.031(0.991, 1.072)	0.963(0.887, 1.044)
34 °C Lag 21	1.057(1.012, 1.103) *	0.987(0.904, 1.079)
34 °C Lag 28	1.001(0.939, 1.066)	1.050(0.926, 1.191)

* *p* < 0.05.
